# Supplementary material for: Generation of Stable Epithelial–Mesenchymal Hybrid Cancer Cells with Tumorigenic Potential
Source: Cancers (Basel). 2023 Jan 22;15(3):684. doi: 10.3390/cancers15030684 (PMC9913490; doi:10.3390/cancers15030684)
Supplement: Supplementary file 1 [file cancers-15-00684-s001.zip › Supplementary Figures.pdf]

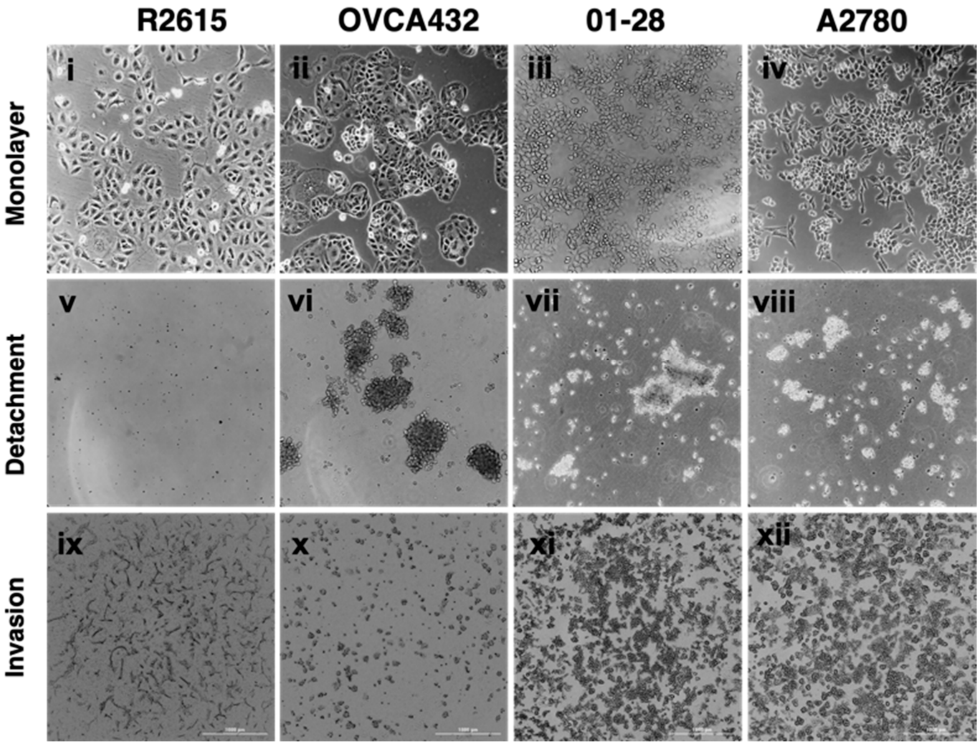

Supplementary Figure S1. Characterization of the epithelial–mesenchymal biological phenotypes in ovarian cancer cell lines. Cellular images shown as (i–**iv**) monolayer. The EMT biological phenotype characterizations were conducted by (**v**–**viii**) anoikis, and (**ix**–**xii**) invasion assays. The ovarian cancer cell lines shown are (**i**, **v**, **ix**) R2615, (**ii**, **vi**, **x**) OVCA432, (**iii**, **vii**, **xi**) 01-28, and (**iv**, **viii**, **xii**) A2780.

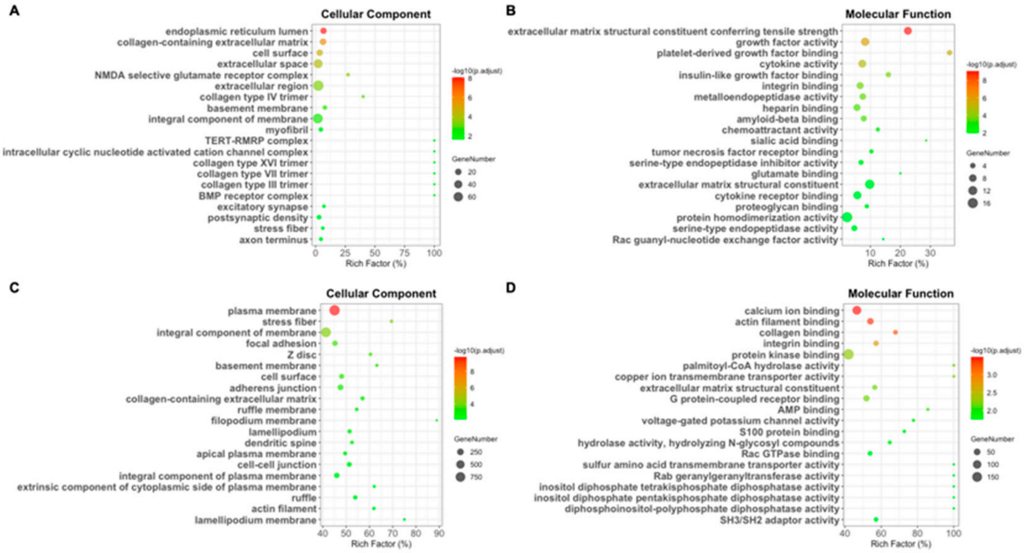

Supplementary Figure S2. GO enrichment analysis of the E/M hybrid state and mesenchymal cells. (**A**) Top 20 significant GO enrichment terms for cellular components in the E/M hybrid compared to the epithelial cells. (**B**) Top 20 significant GO enrichment terms for molecular functions in the E/M hybrid compared to the epithelial cells. (**C**) Top 20 significant GO enrichment terms for cellular components in the mesenchymal-to-epithelial cells. (**D**) Top 20 significant GO enrichment terms for molecular functions in the mesenchymal-to-epithelial cells.

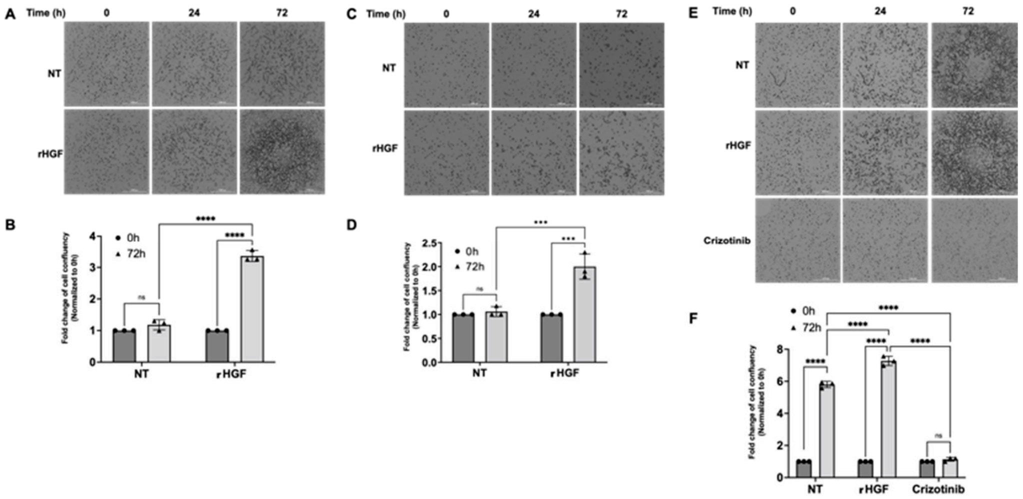

Supplementary Figure S3. Characterization of the effect of HGF on the invasion capacity of R2615, OVCA432, and OVCA433. (**A**) Invasion assay of R2615 (i) 0, (ii) 24, and (iii) 72 h timepoints in the presence or absence of 80 ng/mL rHGF. (**B**) Quantification of the R2615 invasion assay presented as the fold change of cell confluency normalized to cells in 0 h (time of seeding). Bars represent the means ± SEM; p-value ****<0.0001, n.s. = non-significant. Two-way ANOVA was used to define the p-value. (**C**) Invasion assay of OVCA432 (i) 0, (ii) 24, and (iii) 72 h timepoints in the presence or absence of 80 ng/mL rHGF. (**D**) Quantification of the OVCA432 invasion assay presented as the fold change of cell confluency normalized to cells in 0 h (time of seeding). Bars represent the means ± SEM; p-value ***<0.001, n.s. = non-significant. Two-way ANOVA was used to define the p-value. (**E**) Invasion assay of OVCA433 (i) 0, (ii) 24, and (iii) 72 h timepoints in either the presence or absence of 80 ng/mL rHGF or the addition of c-MET inhibitor, 100 nM Crizotinib. (**D**) Quantification of the OVCA433 invasion assay presented as the fold change of cell confluency normalized to cells in 0 h (time of seeding). Bars represent the means ± SEM; p-value ****<0.0001, n.s. = non-significant. Two-way ANOVA was used to define the p-value.

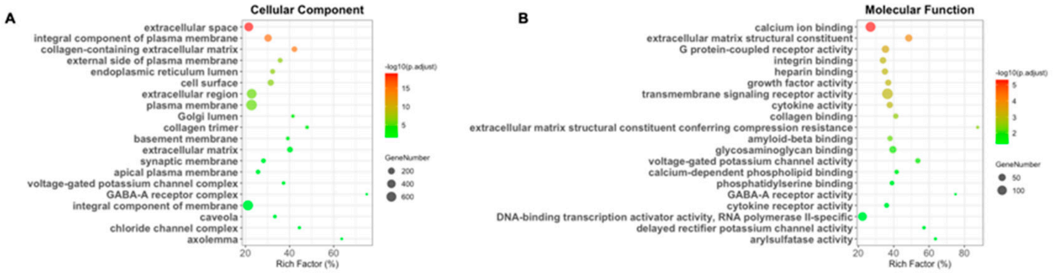

Supplementary Figure S4. GO enrichment analysis of the HGF-educated E/M hybrid state. (**A**) Top 20 significant GO enrichment terms for cellular components in HGFR182 compared to R182. (**B**) Top 20 significant GO enrichment terms for molecular functions in HGR182 compared to R182.
