# Supplementary material for: Generation of Stable Epithelial–Mesenchymal Hybrid Cancer Cells with Tumorigenic Potential
Source: Cancers (Basel). 2023 Jan 22;15(3):684. doi: 10.3390/cancers15030684 (PMC9913490; doi:10.3390/cancers15030684)
Supplement: Supplementary file 1 [file cancers-15-00684-s001.zip › Table S1 Antibody list.pdf]

Supplementary Table S1. **Antibodies used for western blot analyses.**

Antibodies	Dilution	Catalog no. & Manufacturer
Anti-Twist1	1:500	CS-81417, Santa Cruz Biotechnology, Dallas, TX
Anti- SOX2	1:1000	3579, Cell Signaling Technology, Danvers, MA
Anti-β-catenin	1:1000	8480, Cell Signaling Technology, Danvers, MA
Anti-E-cadherin	1:1000	14472, Cell Signaling Technology, Danvers, MA
Anti-Claudin3	1:1000	83609, Cell Signaling Technology, Danvers, MA
Anti-N-cadherin	1:1000	13116, Cell Signaling Technology, Danvers, MA
Anti-Vimentin	1:1000	5741, Cell Signaling Technology, Danvers, MA
Anti-Slug	1:1000	9585, Cell Signaling Technology, Danvers, MA
anti-phospho-c-MET	1:1000	3077, Cell Signaling Technology, Danvers, MA
anti-c-MET	1:1000	8198, Cell Signaling Technology, Danvers, MA
anti-phospho-ERK1/2	1:1000	4370, Cell Signaling Technology, Danvers, MA

anti-ERK1/2	1:1000	9102, Cell Signaling Technology, Danvers, MA
anti-tenascin-C	1:1000	33352, Cell Signaling Technology, Danvers, MA
anti-GAPDH	1:10000	G8795, Sigma, St. Louis, MO
peroxidase-conjugated anti-rabbit IgG	1:10000	Dako, Glostrup, Denmark
peroxidase-conjugated anti-mouse IgG	1:10000	Dako, Glostrup, Denmark
