# Supplementary material for: Generation of Stable Epithelial–Mesenchymal Hybrid Cancer Cells with Tumorigenic Potential
Source: Cancers (Basel). 2023 Jan 22;15(3):684. doi: 10.3390/cancers15030684 (PMC9913490; doi:10.3390/cancers15030684)
Supplement: Supplementary file 1 [file cancers-15-00684-s001.zip › Table S2 Primer list.pdf]

Supplementary Table S2. **Primers used for qRT-PCR analyses.**

**Gene**	**Forward (5'-3')**	**Forward (5'-3')**
HGF	ACGCTACGAAGTCTGTGA	AAGAATTTGTGCCGGTGT
TNC	TCTTGAAGGCAGGCGCAAAC	CCAAATGCCCAGGTGTGGACCGAT
VIM	ATTCCACTTTGCGTTCAAGG	CTTCAGAGAGAGGAAGCCGA
KRT18	AAATCCGGGAGCACTTGGAG	CAATCTGCAGAACGATGCGG
EPCAM	GTGCTGGTGTGTGAACACTG	GAAGTGCAGTCCGCAAACTT
SOX2	AGCTACAGCATGATGCAGGA	GGTCATGGAGTTGTACTGCA
ZO1	CAGAACCAAAGCCTGTGTATG	TTAGGTAGGACACCATCAGATGGA
TWIST1	GTCATGGCCAACGTGCGGGA	GCCGCCAGCTTGAGGGTCTG
MMP1	CAGAGATGAAGTCCGGTTTTTC	GGGGTATCCGTGTAGCACAT
MMP3	AAATCCTGATCTTTAAAGACAGGCACT	GAGATCAAATGCAATTCAGGTTCA
COL6A3	GAGCAGCTTGACAACATTGCC	GCCCAGAGCACTTGCAGG
COL4A1	CCGGATCACATTGACATGAAACC	TGGAAACCAGTCCATGCTCG

c-MET	TGAGAAGGCTAAAGGAAA	TCAAAGGCATGGACATAC
VAV3	ACCAATGGACTGCGAAGAAC	GCTTGACTGCATCACTTGGA
GAPDH	TGACGCTGGGGCTGGCATT	GGCTGGTGGTCCAGGGGT
